# The role of family and peer factors in the development of early adolescent depressive symptoms: A latent class growth analysis

**DOI:** 10.3389/fpsyt.2022.914055

**Published:** 2022-09-15

**Authors:** Jiaying Zhang, Guangyao Lin, Qiaole Cai, Qian Hu, Yuan Xu, Zhaoming Guo, Defan Hong, Yingying Huang, Yijun Lv, Jing Chen, Suo Jiang

**Affiliations:** ^1^The Affiliated Wenzhou Kangning Hospital of Wenzhou Medical University, Wenzhou, China; ^2^Department of Applied Psychology in School of Mental Health, Wenzhou Medical University, Wenzhou, China; ^3^School of Foreign Languages, Wenzhou Medical University, Wenzhou, China; ^4^School of Innovation and Entrepreneurship, Wenzhou Medical University, Wenzhou, China; ^5^Planning and Development Decision Institute (Higher Education Research Institute), Wenzhou Medical University, Wenzhou, China

**Keywords:** depressive symptoms, early adolescence, development trajectories, family, peers

## Abstract

Few studies have explored the trajectories of Chinese early adolescent depressive symptoms or comprehensively considered the factors of family and peers. The present study aimed to identify the trajectories of depressive symptoms in early adolescence using a school-based sample assessed in three waves. The study also examined whether family and peer factors were significant predictors. A total of 586 Chinese primary and middle school students participated in the survey. A growth mixture model was used to find the trajectories of depressive symptoms, and multinominal logistic regression was used to identify the predictors. Three trajectories were identified, including a stable-low class, an increasing class, and a high-decreasing class. Results indicated that gender, parental psychological aggression and neglect, parental psychological control, traditional bullying/cyberbullying victimization, and friendship quality were significant predictors. However, witnessing intimate partner violence, parental behavior control, and traditional bullying/cyberbullying perpetration could not significantly predict the trajectories. The findings of this study can provide an empirical basis for teachers and clinical interveners to determine different development trajectories of depressive symptoms and carry out prevention and intervention.

## Introduction

Adolescence is a key period in individual physical and mental development. It is also a period in which depression can easily occur (1). A meta-analysis found that the detection rate of Chinese adolescent depressive symptoms was 28.4% (2). Depressive symptoms have an important impact on the academic performance of adolescents, their social adaptation, and their physical/mental health (3). The high detection rate and negative effects of depressive symptoms show the necessity to study their occurrence and development. Recently, an emerging body of research has paid more attention to heterogeneity in the development of adolescent depressive symptoms and the factors of family, peers, and other aspects (4–6). However, few studies have explored the trajectories of Chinese early adolescent depressive symptoms or considered the factors of family and peers comprehensively. The present study aimed to identify the different trajectories of depressive symptoms during early adolescence, recognize the family and peer predictors, add new evidence for empirical research, and provide practice guidance.

### The heterogeneity of the development of adolescent depressive symptoms

Heterogeneity and individual differences exist in the occurrence and development of adolescent depressive symptoms (7). Person-centered approaches (such as cluster analysis, latent class analysis, and latent growth mixture modeling) focus on the relationships among individuals, and the goal is to classify individuals into distinct groups or categories based on individual response patterns so that individuals within a group are more similar than individuals between groups. Latent class growth analysis (LCGA) is a special type of latent growth mixture modeling (LGMM), whereby the variance and covariance estimates for the growth factors within each class are assumed to be fixed to zero. By this assumption, all individual growth trajectories within a class are homogeneous. The benefit of this approach is the identification of distinct classes prior to conducting LGMM. It serves as a starting point for conducting LGMM. In terms of computation, it is easy to specify in Mplus and the zero constraints on the variance estimates allow for faster model convergence (8). Many researchers used LCGA to identify the heterogeneous trajectories of different classes of adolescent depressive symptoms (4–6). These results supported the trajectories of three or four classes. A meta-analysis of 18 studies on the developmental trajectories of adolescent depressive symptoms showed that a four-class trajectory was the best-fitting model: ongoing stable-low levels of depressive symptoms; very high depressive symptoms initially, but a steep decrease in symptoms over time; moderately high depressive symptoms initially, but symptoms decreasing over time; initially low levels of symptoms increasing over time (5). The latest study on Chinese early adolescents identified four trajectories of depressive symptoms: low stability, low growth, medium growth, and medium reduction (6). Among the four different trajectories, some classes, such as low stability, increasing, and decreasing to some extent exist stably. Previous studies have confirmed that most adolescents are characterized by low and stable depressive symptoms (5, 6, 9–11). Some studies suggest that depressive symptoms tend to increase slowly in early adolescence (usually 10–14 years old) (12, 13), and such adolescent subgroups were also confirmed in the later LCGA studies (11). In addition, many studies have found that a small number of adolescents show the characteristics of decreasing depressive symptoms, which cannot be ignored (14–17). Other LCGA studies also identified these three classes: stably low class, increasing class, and high-decreasing class (9, 18–21). Based on previous studies, we hypothesized three classes in the developmental trajectories of Chinese early adolescent depressive symptoms: stably low class, increasing class, and high-decreasing class.

### Predictors of the development of adolescent depressive symptoms

The development of adolescent depressive symptoms has different trajectories, which may be related to a series of individual and environmental factors. Gender is a key predictor of depressive symptoms. Gender differences in vulnerability to depressive symptoms in adolescence have been widely investigated (11, 22). The consensus is that females are more susceptible to depressive symptoms in adolescence (11, 23). The diathesis-stress model is an integrated perspective explaining the occurrence of diseases. The model shows that adolescents have innate susceptibility through genetic, endocrinal, biochemical, or environmental factors. These factors interact with psychological and social stressors to produce physical and mental problems (24). Some scholars believe that a genetic history of depression and psychosocial stressors are strong predictors of adolescent depression (3). The vast majority of adolescents with depression have experienced long-term social and psychological pressure, such as discord in family relations or disharmony in their parents’ marriage, domestic violence, abuse, and neglect and school difficulties (such as bullying and academic failure), and social isolation (25). This kind of exposure to high levels and various forms of victimization was called poly-victimization, having even more harmful and less reversible effects on victims (26). The relation between poly-victimization and adolescent depressive symptoms has also been confirmed in Chinese culture (27–29). In addition, according to the interpersonal theory of depression (7), negative interpersonal experience makes individuals tend to form more negative cognitive schema and self-evaluation, and deterioration of self-cognition caused by negative interpersonal experiences will continue to increase, eventually increasing the risk of depression (6). In early adolescence, in particular, individuals face a major process of psychological development during which they gradually separate from their parents and become more affected by their peers. Therefore, while individuals are affected by some family factors, peer factors play an increasingly important role.

#### Family predictors on the development of adolescent depressive symptoms

The family environment has a far-reaching impact on adolescent psychosocial development (30). Chinese culture also holds that family serves as a critical influencing aspect of depression (31). Some researchers have summarized the family factors that increase the risk of adolescent depression as follows: long-term family stress, negative parenting behaviors, low-level family support and attachment, neglect, and parents’ marital conflict (32). Conflicts in the family environment can disrupt the core sense of security requisite for children’s adaptive coping responses to stress so that increases the risk of depression (33, 34). It is believed that exposing children to violence between parents is a form of psychological abuse (35). An empirical study found that exposure to parents’ aggression in marital conflicts (also called intimate partner violence) was a risk factor for adolescent depression (36). Witnessing is one of the major ways adolescents become exposed to intimate partner violence (37). A study has shown that witnessing intimate partner violence can positively predict depressive symptoms of Chinese college students (38). However, the effect of witnessing intimate partner violence on adolescent depressive symptoms was paid little attention in Chinese culture. More importantly, negative parent-child interactional factors, such as poor parenting style, parental emotional abuse, and neglect also have an important effect on the development of individual adaptability. A meta-analysis showed that parental emotional abuse and neglect were significantly and positively correlated with adolescent depressive symptoms (39), which could continue into adulthood (40). A large number of studies have found that parental emotional abuse and neglect increased the risk of adolescent depression in Chinese culture (41–44). Regarding parenting styles, parental control is the core content, including parental behavior control and psychological control (45). Parental behavior control usually includes openly regulating adolescent behaviors, such as establishing rules and boundaries, developing consistent discipline, and monitoring adolescent activities. However, psychological control is different. It refers to a manipulated parenting strategy in which parents aim to force adolescents to think and act in the same way as themselves (46). Parental psychological control interferes with independent adolescent development and causes continuous dependence (47), which was identified as a strong predictor of adolescent depression (48–50). A longitudinal study investigated the development trajectories of parental psychological control perceived by adolescents, similar to the trajectories of adolescent depressive symptoms. It further indicated that parental psychological control was closely related to adolescent depressive symptoms (51). However, the relationship between parental behavior control and adolescent depressive symptoms has received less attention. Previous studies have a unified view that parental psychological control is more closely related to adolescent internalized problems (mainly depression and anxiety), while parental behavior control is more closely related to adolescent externalized problems (mainly bad behavior) (52). A later study found that parental behavior control significantly negatively predicted adolescents’ depression, but this effect was masked by adolescents’ personality traits (53). Parental behavior control often reflects parental care and love, which is often accompanied by positive reinforcement and verbal guidance. It may promote the improvement of adolescent psychological well-being, thus providing a buffer for the occurrence and development of depressive symptoms (54). A cross-cultural study also pointed out that in a culture where family intimacy is particularly valued, parental behavior control might predict less internalized problems in adolescence (55). Chinese culture places great emphasis on mutual support and intimacy between family members, where parental behavioral control might be a protective factor against depressive symptoms. The effect of parental behavioral control is not simply linear and only a moderate degree is the best (56). In short, witnessing intimate partner violence, parental psychological aggression/neglect, and psychological control were essential family factors that were closely related to adolescent depressive symptoms. We hypothesized that these family factors could predict the development of adolescent depressive symptoms.

#### Peer predictors of the development of adolescent depressive symptoms

The supporting role and the potentially negative impact of peer relationships become evident in adolescence. Low level of peer attachment, peer rejection, and peer interaction related to victimization are key factors for adolescent depressive symptoms (57). Bullying is a common adverse peer factor, and it is a particular type of aggressive behavior. There is an imbalance of power between the parties involved (the victim has difficulties protecting themself from bullying), and it is repetitive and deliberate (58). Traditionally, bullying comes in three forms: verbal bullying, physical bullying, and relational bullying (58). Nowadays, bullying also occurs in cyberspace in the form of cyberbullying (59). As a new type of bullying, cyberbullying is destructive and extreme because it can happen anytime and anywhere, and the content of cyberbullying remains accessible after the event, which brings repeated harm to the victims (60). Previous studies have found that traditional bullying perpetration/victimization (61) and cyberbullying perpetration/victimization (62) were related to the increase in adolescent depressive symptoms. A cross-cultural study shows that bullies (during adolescence) in China had more depressive symptoms in adulthood compared to victims, which emphasizes the impact of bullying (63). Adolescents who bully others were found to have more psychological and physical problems than their peers and had an increased risk for depression (64). A recent study also found that both bullying perpetration and victimization were risk factors for adolescent depressive symptoms (65). Concurrently, researchers generally believe that high-quality interpersonal relationships have a significant protective effect on the occurrence and development of adolescent depressive symptoms (3). Adolescents can gain a sense of security, emotional support, and self-confidence in their interactions with friends (66). High friendship quality plays a protective role in the relationship between early childhood adverse experiences and depression (67). At the early stage, some researchers believed that friendship quality had a direct impact on adolescent depressive symptoms (68). Later, some longitudinal studies found that friendship quality had a significant negative prediction on adolescent depressive symptoms (57, 69). High friendship quality is not only the basis for the healthy development of adolescents but can also compensate, to a certain extent, for a bad family environment (70). In short, traditional bullying perpetration/victimization and cyberbullying perpetration/victimization were essential factors in the impact of peers, and they were closely related to adolescent depressive symptoms. We hypothesized these peer factors could predict the development of adolescent depressive symptoms.

### The current study

The present study assessed a school-based sample of adolescents in three waves and conducted a GMM. We used multinominal logistic regression analysis to examine whether family (witnessing intimate partner violence, parental psychological aggression, and neglect, parental psychological and behavioral control) and peer (traditional bullying perpetration and victimization, cyberbullying perpetration, and victimization, friendship quality) factors would significantly predict adolescent depressive symptom trajectories.

## Materials and methods

### Participants and procedures

As part of a large data collection, data were collected in three waves (half-yearly) from primary and middle school students in eight schools in a city in China. In this study, stratified cluster random sampling was used. Students from eight public and private schools in Grade 4, 5, and 7 were selected by class to participate in the survey. A total of 620 participants were selected after the baseline measurement (T1: April 2017). The second measurement was conducted in October 2017 (T2), and the third measurement was conducted in April 2018 (T3). 586 participants participated in the survey three times (the invalid participants failed to complete three tests together due to transfer, examination during the test, or student activities). The loss rate was 3.7%. Among the participants, 47.8% were females. The average age was 11.73 years old (*SD* = 1.33), ranging from 10 to 14 years. Monthly household income was on a seven-point scale (1 = ￥1,500; 2 = ￥1,500–3,000; 3 = ￥3,000–5,000; 4 = ￥5,000–8,000; 5 = ￥8,000–12,000; 6 = ￥12,000–20,000; and 7 = > ￥20,000). Parent education was on a four-point scale (1 = did not complete middle school; 2 = high school; 3 = college diploma or trades certificate; and 4 = university undergraduate degree and more). The average monthly household income level of participants was 4.73. The average education level of the parents of the participants was 1.82. All subjects gave written informed consent by the Declaration of Helsinki. They were free to withdraw during the research process, and their privacy and personal information were respected and kept confidential.

### Measures

#### Depressive symptoms

The Chinese Version of the Center for Epidemiologic Studies Depression Scale was used to measure past-week depressive symptoms (71). It contains 20 items, including the four dimensions of depressed affect, positive affect, somatic and retarded activities, and interpersonal factors. The scale requires participants to evaluate the frequency of corresponding symptoms within a week (e.g., “I’m worried about some small things”). Responses were given on a four-point frequency scale. 1 = “almost never (less than once a day)” to 4 = “most of the time (5–7 days).” A higher mean score indicates more depressive symptoms. The questionnaire had good reliability and validity when used with Chinese adolescents (72). In the present study, the Cronbach’s α for all items of each wave were 0.87, 0.88, and 0.90, respectively.

#### Witnessing intimate partner violence

The Chinese version of the revised Conflict Tactics Scales Questionnaire (73) was used to measure witnessing intimate partner violence. We used 25 of the items (the item about gun use was deleted for cultural reasons). We included the three dimensions of psychological aggression, physical violence, and injuries. Each item was responded to on a seven-point scale ranging from 1 (never) to 7 (almost every day). Participants were asked to use the scale to answer the frequency of witnessing violence between their father (or stepfather) and mother (or stepmother) (e.g., “witness their father/mother yelling at their partner”). After reversing the items, the average score of all items was calculated. Higher scores indicated higher frequencies of witnessing intimate partner violence. CFA was conducted because it was the first time this questionnaire was used to measure Chinese adolescents witnessing intimate partner violence. The three-factor model provided a good fit for the data (χ^2^/*df* = 3.43, RMSEA = 0.06, CFI = 0.93, TLI = 0.91, SRMR = 0.03). In the present study, Cronbach’s α was 0.95 in the first wave.

### Parental psychological aggression and neglect

A validated Chinese version of the parent-child Conflict Tactics Scale was used to measure parental psychological aggression and neglect (74, 75). Two subscales were selected: psychological aggression (e.g., “father/mother yelled at you”) and neglect (e.g., “even if your parents think you should be accompanied by adults, your father/mother still left you at home alone”), a total of 10 items. Each item was responded to on a seven-point scale ranging from 1 (never) to 7 (almost every day). After reversing the items, the higher mean score indicated that the individual suffers from more psychological aggression and neglect. The two-factor model provided a good fit for the data (χ^2^/df = 2.81, RMSEA = 0.04, CFI = 0.92, TLI = 0.91, SRMR = 0.04). In the present study, Cronbach’s α was 0.83 in the first wave.

### Parental psychological control

The Chinese version of the Parental Control Questionnaire (76) was used to measure parental psychological control. Adolescents rated 18 items on a five-point scale, including the dimension of guilt induction (e.g., “My parents tell me that I should feel guilty when I do not meet their expectations”), love withdrawal (e.g., “My parents act cold and unfriendly if I do something they do not like”), and authority assertion (e.g., “My parents tell me that what they want me to do is the best for me and I should not question it”), ranging from 1 (not at all true) to 5 (very true). The higher mean score indicated a higher level of parental psychological control. In the present study, Cronbach’s α was 0.94 in the first wave.

### Parental behavioral control

The Chinese version of the Parental Control Questionnaire (76) was used to measure parental behavioral control. Adolescents rated 16 items on a five-point scale, including two dimensions: solicitation (e.g., “My parents initiate a conversation with me about what happens during my free time”) and restriction (e.g., “my parents require me to ask for their permission before I go out after school”), ranging from 1 (never) to 5 (every day). The higher mean score indicated a higher level of behavior control. In the present study, Cronbach’s α was 0.91 in the first wave.

### Traditional bullying perpetration and victimization

The Chinese version of the School Bullying Perpetration and Victimization Scale was used (58, 77). The scale contains seven items for bullying perpetration (e.g., “I deliberately hit, kick, push or bump into others”). The subscale of bullying victimization also includes seven items (e.g., “I was teased or made fun of by others”). Items were rated on a five-point scale, with response options ranging from 0 (never) to 4 (five or more times). Mean scores were calculated with higher scores representing more severe bullying perpetration or victimization. In the present study, the Cronbach’s α of the perpetration subscale and victimization subscale were 0.81 and 0.82, respectively, in the first wave.

### Cyberbullying perpetration and victimization

Cyberbullying was assessed using nine items from the Chinese version of the Cyberbullying Scale (78). The scale consists of nine items for perpetration (e.g., “tease others in a meaningful way online or through text messages”) and, correspondingly, nine items for victimization (e.g., “I was teased by others in a meaningful way online or through text messages”). The participants rated all items ranging from 1 (never) to 5 (almost always). The average scores were calculated, with the higher scores representing a higher rate of cyberbullying perpetration and victimization. In the present study, the Cronbach’s α of the perpetration scale and victimization scale were 0.89 and 0.92, respectively, in the first wave.

### Friendship quality

The Chinese version of the Friendship Quality Questionnaire was used (79, 80). It contains 18 items on the five dimensions of validation/caring, peers/recreation, help/guidance, intimate exchange, and conflict/betrayal. The items described the relationship with the best friend (e.g., “My best friend likes me even if others don’t like me”). Participants responded using a four-point scale, ranging from 1 (not at all true) to 4 (really true). Mean scores were calculated, with higher scores indicating higher levels of friendship quality. In this study, the Cronbach’s coefficient α was 0.83 in the first wave.

### Data analysis

Descriptive statistics and correlation analysis were performed using SPSS 19.0. LCGA was constructed in Mplus8.3 using the construction procedure proposed by Jung and Wickrama (8) to investigate the trajectories of adolescent depressive symptoms. Multinominal logistic regression was used to explore the family and peer predictors on the trajectories. The variables were included in the same regression model. Missing values were processed by Maximum Likelihood Estimation.

### Test of common-method biases

In the present study, the common-method biases were controlled by anonymous measurement and reverse scoring of some items (81). CFA was used to test the common-method biases of all self-evaluation items. The results showed that the single-factor model fitting was very poor (χ^2^/df = 9.25, CFI = 0.37, TLI = 0.35, RMSEA = 0.09, SRMR = 0.14), so there were no common-method biases.

## Results

### Preliminary analysis

Table 1 presents the descriptive statistics for all the variables and the correlations among the variables. As Table 1 shows, witnessing intimate partner violence, parental psychological control, parental psychological aggression and neglect, traditional bullying perpetration and victimization, cyberbullying perpetration, and victimization were significantly and positively correlated with baseline depressive symptoms. Parental behavior control and friendship quality were significantly and negatively correlated with baseline depressive symptoms.

**TABLE 1:** Descriptive statistics of study variables and correlations between them.

	*M* ± *SD*	1	2	3	4	5	6	7	8	9
1. Baseline depressive symptoms	1.71 ± 0.50									
2. Witnessing intimate partner violence	1.23 ± 0.50	0.31**								
3. Psychological aggression/neglect	1.80 ± 0.86	0.41**	0.44**							
4. Parental psychological control	2.06 ± 0.84	0.21**	0.23**	0.46**						
5. Parental behavioral control	2.91 ± 0.86	−0.13**	0.04	0.01	0.29**					
6. Traditional bullying perpetration	0.39 ± 0.58	0.27**	0.28**	0.41**	0.24**	–0.07				
7. Traditional bullying victimization	0.76 ± 0.84	0.40**	0.27**	0.35**	0.23**	–0.06	0.33**			
8. Cyberbullying perpetration	1.19 ± 0.37	0.25**	0.23**	0.26**	0.20**	–0.02	0.28**	0.24**		
9. Cyberbullying victimization	1.25 ± 055	0.27**	0.31**	0.30**	0.27**	0.00	0.25**	0.24**	0.39**	
10. Friendship quality	2.53 ± 0.30	−0.23**	−0.15**	−0.15**	–0.04	0.26**	−0.24**	−0.26**	−0.18**	−0.20**

*p < 0.05, **p < 0.01, ***p < 0.001.

### Identification of depressive symptom trajectories

According to the construction procedure proposed by Jung and Wickrama (8), an unconditional latent growth curve model was first fitted to the data. A cubic growth model, with quadratic and cubic variances fixed to zero to achieve a positive definite psi matrix, was used as a basis for subsequent analyses. The model provided good overall fit (χ^2^/*df* = 5.10, *p* = 0.020; RMSEA = 0.08, CFI = 0.99, TLI = 0.98, SRMR = 0.02). However, there were significant variances for the intercept (σ^2^ = 60.12, SE = 6.46, *p* < 0.001) and linear slope (σ^2^ = 5.86, SE = 2.98, *p* = 0.021), indicating that there were systematic differences in the trajectories of adolescent depressive symptoms among individuals. The single-class model did not adequately account for variation in adolescent depressive symptoms trajectories.

Next, GMMs of 1–5 classes were used to explore subgroups with distinct symptom trajectories. For GMM models, means and slopes (linear, quadratic, and cubic) were allowed to vary between classes, and means and linear slopes were allowed to vary within classes, with quadratic and cubic slope variances fixed to zero within classes for model estimation purposes. Lower AIC, BIC, and adj. BIC values are indicative of a better model fit. Significant VLMR and BLRT tests favor the k class model over the k-1 class model. Higher entropy and latent class probabilities indicate greater parsimony and classification accuracy [Jung and Wikrama (8); Hill et al. (4)]. Table 2 shows the relative fit indices and numbers of people in each subgroup for all models.

**TABLE 2:** Relative fit indices for class models 1 through 5.

Number of classes	AIC	BIC	Adj. BIC	BLRT	VLMR	Entropy	*N*	Percentage
1	11616.36	11629.48	11619.96	−	−	−	−	
2	11578.00	11608.62	11586.39	*p* = 0.030	*p* = 0.038	0.40	137/449	23%/77%
**3**	**11539.52**	**11587.63**	**11552.71**	***p* < 0.001**	***p* < 0.001**	**0.75**	**461/95/30**	**78%/16%/6%**
4	11529.51	11595.11	11547.49	*p* < 0.001	*p* = 0.168	0.79	465/16/38/67	79%/3%/6%/12%
5	11537.69	11620.78	11560.47	*p* < 0.001	*p* = 0.216	0.80	468/15/65/38/0	80%/3%/11%/6%/0

Bold shows the best fit.

Adjusted BIC indicated that the four-class model was better. VLMR showed a preference for the two-class or three-class model. BLRT value indicated that two-class and above models was better. Entropy indicated that 3-, 4-, and 5-class models were all acceptable. Although four-class and five-class models showed higher classification accuracy, VLMR were not significant, indicating that the models were not well-fitted. Therefore, the three-class model was retained.

**FIGURE 1:**
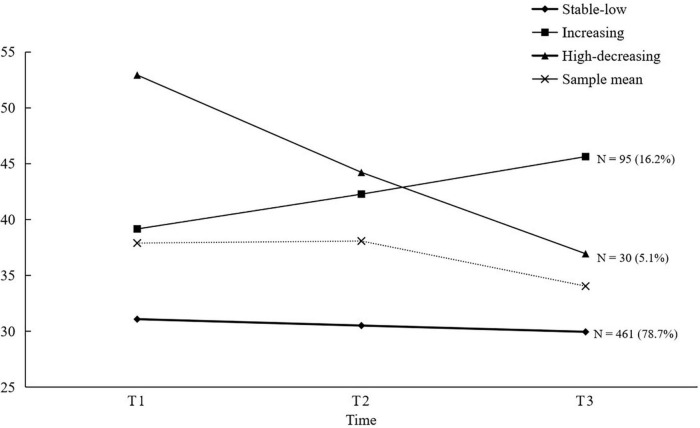
Depressive symptoms trajectories.

Figure 1 shows the three classes of depressive symptom trajectories. The first class (*n* = 461) showed stable and mild depressive symptoms, and the baseline depressive symptoms were low. It was named **the stable-low class**. The intercept (3.43, SE = 0.015, *p* < 0.001) and linear slope were significant (−0.019, SE = 0.007, *p* = 0.008). The second class (*n* = 95) showed almost the same baseline depressive symptoms as the average but showed increasing depressive symptoms over time. It was named **the increasing class**. The intercept (3.65, SE = 0.043, *p* < 0.001) and linear slope were significant (0.076, SE = 0.020, *p* < 0.001). The baseline depressive symptoms of the third class (*n* = 30) were significantly higher than the average. Although there were decreased depressive symptoms over time, the depressive symptoms during the three measurements were higher than the average. It was named **the high-decreasing class.** The intercept (3.96, SE = 0.006, *p* = 0.000) and linear slope (−0.18, SE = 0.030, *p* < 0.001) were significant.

### Predictors of depressive symptoms trajectories

Multinomial logistic regression was adopted to investigate the predictors of family (witnessing intimate partner violence, parental psychological aggression and neglect, parental psychological control, and behavioral control) and peer (traditional bullying perpetration and victimization, cyberbullying perpetration and victimization, and friendship quality) variables on the trajectories of adolescents’ depressive symptoms. Demographic variables and predictors for the base time were taken as independent variables with different depressive symptoms trajectories as the dependent variables. Table 3 shows the results. First, to make a comprehensive comparison between the classes, the stable-low class was taken as the reference group. The results showed that compared with the increasing class, boys were more likely to appear in the stable-low class (OR = 0.448). The higher the parental psychological aggression and neglect (OR = 1.734), traditional bullying victimization (OR = 1.535), and cyberbullying victimization (OR = 1.825), the more likely adolescents were to appear in the increasing class. Compared with the high-decreasing class, the higher parental psychological control (OR = 1.611), traditional bullying victimization (OR = 1.595), and cyberbullying victimization (OR = 1.806), the more likely adolescents were to appear in the high-decreasing class. The higher the quality of friendship, the more likely adolescents were to appear in the stable-low class (OR = 0.316). Then, taking the increasing class as the reference, the results showed that the higher the parental psychological control, the more likely adolescents were to appear in the high-decreasing class (OR = 1.793).

**TABLE 3:** Predictors from family and peers of depressive symptom trajectories.

Predictors	Stable-low class *vs.* increasing class			Stable-low class *vs*. high-decreasing class			Increasing class *vs*. high-decreasing class		
	*P*	OR	95% CI	*P*	OR	95% CI	*P*	OR	95% CI
Gender (1 = male)	**0.007**	**0.448**	**0.250∼0.801**	0.683	1.245	0.435∼3.567	0.074	2.783	0.907∼8.540
Origin (1 = urban)	0.292	1.396	0.751∼2.597	0.776	0.859	0.301∼2.452	0.389	0.616	0.204∼1.858
Father’s education	0.420	1.190	0.779∼1.818	0.673	1.169	0.566∼2.414	0.963	0.982	0.452∼2.134
Mother’s education	0.158	0.714	0.447∼1.139	0.868	0.935	0.421∼2.072	0.535	1.310	0.559∼3.070
Witnessing intimate partner violence	0.795	1.061	0.678∼1.660	0.809	1.087	0.551∼2.147	0.908	1.054	0.432∼2.572
Psychological aggression/neglect	**0.000**	**1.734**	**1.275∼2.395**	0.110	1.440	0.920∼2.253	0.167	0.639	0.339∼1.206
Parental behavioral control	0.918	0.984	0.732∼1.326	0.290	0.767	0.470∼1.253	0.662	0.870	0.0464∼1.628
Parental psychological control	0.460	1.127	0.821∼1.546	**0.034**	**1.611**	**1.037∼2.503**	**0.049**	**1.793**	**1.003∼3.207**
Traditional bullying perpetration	0.672	0.909	0.585∼1.413	0.472	0.786	0.408∼1.515	0.491	0.698	0.251∼1.942
Traditional bullying victimization	**0.005**	**1.535**	**1.141∼2.062**	**0.040**	**1.595**	**1.022∼2.490**	0.441	1.240	0.717∼2.145
Cyberbullying perpetration	0.859	1.059	0.566∼1.980	0.983	0.989	0.377∼2.598	0.997	0.997	0.253∼3.934
Cyberbullying victimization	**0.004**	**1.825**	**1.212∼2.749**	**0.029**	**1.806**	**1.064∼3.068**	0.512	0.809	0.429∼1.525
Friendship quality	0.476	0.768	0.372∼1.586	**0.040**	**0.316**	**0.105∼0.951**	0.206	0.383	0.087∼1.694

Bold shows the significant results. OR is odds ratio. If the OR value > 1, the stronger the factor, the less likely the individual is to be in the reference group; On the contrary, if the OR value < 1, it means that the stronger the factor is, the more likely the individual is in the reference group.

## Discussion

This study identified trajectories of depressive symptoms in early adolescence. Most adolescents belong to the stable-low class, a few adolescents belong to the increasing class, and the least number of adolescents belongs to the high-decreasing class. This study also found some significant family and peer predictors. Specifically, parental psychological aggression/neglect, parental psychological control, traditional bullying/cyberbullying victimization, and friendship quality were significant predictors. Based on the investigation of the short-term trajectories of depressive symptoms in early adolescence, this study is the first to examine how family and peer factors are related to the development of depressive symptoms in Chinese adolescents. The results of this study provide new evidence for the development and factors of depressive symptoms in Chinese adolescents.

First, in the trajectories of early adolescent depressive symptoms, the majority of adolescents showed the characteristics of stable and low depressive symptoms. Their depressive symptoms were below the average level and did not fluctuate in follow-ups. The result was consistent with previous findings. The second class of adolescents showed elevated depressive symptoms. The characteristic of this class was that the baseline level was close to the average level but gradually became higher than the average level. In the last measurement, this class showed the highest depressive symptoms among the three classes. Almost one-fifth of individuals belong to this kind of development trajectory. Similar results were found in previous studies (20, 21). This kind of adolescent deserves special attention because they might show an important tendency to clinical adolescent depression. The third class of adolescents showed high-decreasing depressive symptoms, which accounted for the smallest number in the population, only 5%. The class showed the highest baseline depressive symptoms level and a decline in development, but the overall level was still higher than the average. This study found the same conclusions as some long-term follow-up studies using short-term data, showing that the development of adolescent depressive symptoms may be stable and continuous. Therefore, this study emphasizes the early identification and classification prevention of adolescent depressive symptoms.

Second, this study found some significant family and peer predictors of the trajectories of depressive symptoms in early adolescence. Specifically, for girls, parental psychological aggression and neglect, and traditional bullying/cyberbullying victimization were the risk factors for adolescents to show the trajectory of increasing depressive symptoms. For girls, the incidence rate of depression in early adolescence increased dramatically (22) to twice that of boys (82). Girls are more likely to show cognitive styles characterized by negative self-evaluation and rumination, and their emotional experience is more profound than that of boys, which makes adolescent women more likely to have depression (13). This study also emphasizes the adverse effect of parental psychological aggression and neglect. Individuals who grow up with bad parent-child interactions are difficult to form a powerful self or a healthy personality. Such adolescents are very vulnerable internally. When facing the huge physical and mental changes in adolescence, they are often unable to cope with it, which leads to depressive symptoms (40, 83). In addition, this study did not identify the impact of witnessing intimate partner violence on the trajectory of adolescent depressive symptoms. This might be related to the low incidence of intimate partner violence in the sample. Regarding peer predictors, contrary to the hypothesis, this study only found that traditional bullying and cyberbullying victimization were risk factors, while traditional bullying and cyberbullying perpetration could not predict the trajectory of depressive symptoms. The result was consistent with some previous research results (4, 64). Bullying victimization is a passive and negative peer interaction, resulting in a series of negative self-cognition and promoting the development of depressive symptoms (10). Meanwhile, bullying perpetration is a more active peer interaction, a way to express hostility and outward aggression. The participants of this study were in early adolescence. Some researchers believed that individuals who bullied others in early adolescence often seemed to be unscathed because their social status and self-concept were significantly better than those who were bullied. These young people were even regarded as positive leaders, with a good sense of humor, high self-esteem, positive early friendship, and popularity (84).

It is noteworthy that in the comparison between the stable-low class and the high-decreasing class, this study found that parental psychological control and traditional bullying and cyberbullying victimization were the risk factors, while friendship quality was the protective factor. Parental psychological control seemed particularly important for adolescents in the high-decreasing class. This study also identified the significant role of parental psychological control in comparing the high-decreasing class and the increasing class, revealing the negative impact of parental psychological control, a form of parenting that destroys the development of autonomy and self-consciousness. The main ways of psychological control are guilt initiation and withdrawal of love (45). Guilt and self-aggression modes were easy to provoke in adolescents under psychological control for a long time. These modes promote the occurrence and development of depressive symptoms (51). This study also highlighted the protective effect of high-quality friendship. A positive peer reaction is a kind of external resource for adolescents, which can regulate the relationship between other risk factors and individual adaptive development, thus protecting the physical and mental health of adolescents (9). Adolescents need to develop good friendships and obtain peer support. However, this study did not find a significant effect of parental behavior control. Previous studies have paid more attention to the effect of parental behavioral control on adolescents’ bad behaviors (such as problematic internet use and aggressive behavior) and found a protective effect (56, 85). Regarding emotions, one study found that parental behavior control has a positive predictive effect on adolescent generalized anxiety (86). As mentioned earlier, the effect of parental behavior control is complex and non-linear. On the one hand, behavior control can make individuals feel cared for and taken care of, on the other hand, it can also make individuals feel restricted and deprived of freedom (87). In short, the results of this study reveal the importance of parent-child interaction in the development of adolescents. Some negative interactions, such as bad parenting style, abuse, and aggression, were closely related to the development of depressive symptoms. This study also identified the effect of bullying victimization and the protective effect of a good peer relationship. To some extent, the bad peer interaction suffered by individuals in adolescence is a repetition of the bad parent interaction. These findings suggest that teachers and psychological workers attach importance to parents’ work (e.g., lectures for parents and parent-child psychological group counseling) and the prevention of bullying. More importantly, peer communication training for adolescents is necessary to better help them master the maintenance strategies of high-quality friendship. When individuals have the consciousness and ability to maintain a good friendship, they will also have more strength to cope with the growing pressure and conflicts.

In conclusion, this study found different trajectories of depressive symptoms in early adolescence and explored the effects of parental psychological aggression and neglect, parental psychological control, traditional bullying/cyberbullying victimization, and friendship quality. The research combined individual-centered and data-centered methods to complement the results in the related fields. The results help to guide interveners to effectively prevent the occurrence and development of adolescent depression from the perspective of family and peer relationships. For example, to decrease adolescent depression, we recommend working with parents, providing mental health education for parents, and calling on parents to interact with their children more healthily. Anti-bullying initiatives should be carried out in schools to prevent bullying.

However, this study has some limitations. First, the follow-up time was too short, and the waves of data were too few. Future research could take place over a longer time and determine a more accurate development trajectory in Chinese culture. Second, this study did not collect the predictors during the three waves. Therefore, it was difficult to obtain dynamic impact results. Future research could investigate the joint trajectory of parental psychological aggression and neglect, parental psychological control, bullying, friendship quality, and depression, to explore in-depth the dynamic impact mechanism. Thirdly, the data in this study were all self-reported. Social desirability might have introduced bias. In future research, multi-source data could be collected to present the development characteristics and influencing factors more objectively and accurately. Finally, the data were collected only in China and the cross-cultural application of the findings needs further consideration, especially for the predictors.

## Conclusion

For individuals in early adolescence, there were three trajectories of depressive symptoms: stable-low class, increasing class, and high-decreasing class. In addition, parental psychological aggression and neglect, parental psychological control, traditional bullying/cyberbullying victimization, and friendship quality were important predictors. These findings could inspire child and youth workers to identify students’ depressive symptom trajectories and carry out targeted prevention and intervention in the context of family and peer factors.

## Data availability statement

The raw data supporting the conclusions of this article will be made available by the authors, without undue reservation.

## Ethics statement

The studies involving human participants were reviewed and approved by The Ethics Committee of Wenzhou Medical University. Written informed consent to participate in this study was provided by the participants’ legal guardian/next of kin.

## Author contributions

SJ and YL contributed to the conception of the study. JZ, GL, and QC collected all data. JZ, YX, ZG, and QH contributed to the first drafting of the manuscript. SJ, JC, DH, and YH contributed to the critical revision of the manuscript. All authors have read and approved the final manuscript..
